# Comparison of Low-Cost Particulate Matter Sensors for Indoor Air Monitoring during COVID-19 Lockdown

**DOI:** 10.3390/s20247290

**Published:** 2020-12-18

**Authors:** Miron Kaliszewski, Maksymilian Włodarski, Jarosław Młyńczak, Krzysztof Kopczyński

**Affiliations:** Institute of Optoelectronics, Military University of Technology, Gen. S. Kaliskiego 2, 00-908 Warsaw, Poland; maksymilian.wlodarski@wat.edu.pl (M.W.); jaroslaw.mlynczak@wat.edu.pl (J.M.); krzysztof.kopczynski@wat.edu.pl (K.K.)

**Keywords:** indoor air, low-cost particle sensors, human activity, aerosol sources, PM

## Abstract

This study shows the results of air monitoring in high- and low-occupancy rooms using two combinations of sensors, AeroTrak8220(TSI)/OPC-N3 (AlphaSense, Great Notley, UK) and OPC-N3/PMS5003 (Plantower, Beijing, China), respectively. The tests were conducted in a flat in Warsaw during the restrictions imposed due to the COVID-19 lockdown. The results showed that OPC-N3 underestimates the PN (particle number concentration) by about 2–3 times compared to the AeroTrak8220. Subsequently, the OPC-N3 was compared with another low-cost sensor, the PMS5003. Both devices showed similar efficiency in PN estimation, whereas PM (particulate matter) concentration estimation differed significantly. Moreover, the relationship among the PM_1_–PM_2.5_–PM_10_ readings obtained with the PMS5003 appeared improbably linear regarding the natural indoor conditions. The correlation of PM concentrations obtained with the PMS5003 suggests an oversimplified calculation method of PM. The studies also demonstrated that PM_1_, PM_2.5_, and PM_10_ concentrations in the high- to low-occupancy rooms were about 3, 2, and 1.5 times, respectively. On the other hand, the use of an air purifier considerably reduced the PM concentrations to similar levels in both rooms. All the sensors showed that frying and toast-making were the major sources of particulate matter, about 10 times higher compared to average levels. Considerably lower particle levels were measured in the low-occupancy room.

## 1. Introduction

Air quality is an important topic in public health studies, environmental protection, and atmospheric research [1]. The negative effects of air pollution on human health have been widely studied in various epidemiological research studies and multiannual observations [2,3,4]. There is a close relationship between particle size and the deposition region of the respiratory tract. Thus, assuming a polydispersed aerosol distribution, particulate matter permeates the respiratory tract as follows: PM_1_—alveoli, PM_2.5_—alveoli and bronchioles, and PM_10_—alveoli, bronchioles, and upper air tracts [5,6,7]. Indoor particulate matter (PM) can include outdoor particles, as well as those of domestic origin, such as anthropogenic constituents, particles of animal origin, fungi, dust, kitchen fumes, candle and cigarette burning emissions, and so on [8]. In addition to the source of origin, PM composition depends on the ambient conditions which can favor bonding with chemical and biological substances. Consequently, adverse health effects can be further triggered by toxicity and allergic reactions [9,10,11]. 

People spend at least 40–50% of their time in their homes and more than 80% indoors [8], where they are subjected to potentially harmful aerosols. During the COVID-19 lockdown, for millions of people around the world, the time spent at their homes reached 100% and lasted for weeks. For many of them, homes became their current lunchrooms, gyms, entertainment places, and workspaces. This has revealed a growing need for air monitoring in occupied houses. 

For about half a century, thousands of studies concerning sources and health aspects of indoor aerosols have been carried out [12,13]. Most studies indicated that combustion and frying are the main sources of indoor air pollution [14]. The penetration of outdoor PM into indoor space has also been identified as an important source of indoor air pollution [15,16]. There is clear evidence that movement or floor vacuuming, due to air agitation, contributes to dust resuspension from various surfaces [17,18,19]. Multiple sensor comparative studies performed parallelly in various residences comprehensively described the main particle sources and people’s exposure to air pollutants [20]. However, pandemic mobility restrictions forced people to stay in their homes. This offered a unique opportunity to conduct experiments in a new reality according to people’s behavior. Moreover, the experiments were permanently supervised and every activity could be followed. 

Traditional PM sampling such as gravimetric or TEOM (Tapered element oscillating microbalance) technology is commonly approved due to high reliability and accuracy. However, these methods are expensive, need maintenance, and their time resolution is limited [21]. Recent developments in laser and light detection technology have allowed air quality sensors to be miniaturized and commercialized. Evaluation studies conducted worldwide have demonstrated the reliability and precision of popular low-cost sensors, although the literature reports are divergent. Therefore, the data produced should be used with care, and data correction is often suggested due to the disagreement with reference devices [14,22,23,24,25]. Comparison to the reference aerosol spectrometer (Grimm 1.108) showed that low-cost sensors, including PMS5003 used in this study, showed different performance than declared by the manufactures and provided a proper response in a limited range of bins [26]. On the other hand, the PMS series sensor showed a higher measurement quality than the Alphasense OPC-N2 (the previous model of the one used in our study) [27]. Contrary to this, Susan et al. demonstrated that OPC-N2 sized particles identically and in good agreement with the reference, earlier mentioned, Grimm 1.108 aerosol monitor [28]. The comparison performed by Bezantakos et al. showed less than 40% difference between the Alphasense OPC-N2 and Grimm 1.109 instruments [29]. Due to the significant discrepancies among the results, it is necessary to conduct further comparative studies using the various instruments. The use of light scattering has become an approved method in the study of aerosol concentration and size distribution. Many low-cost particle counters have emerged on the market in the last few years, and their effectiveness was evaluated in laboratories [30], indoor environments, and field tests [31,32]. Their inevitable advantages are cost-effectiveness, ease of use, accuracy, fast response, and high resolution. The present study demonstrates a comprehensive multiparameter analysis of indoor air.

The research was focused on the assessment of the levels and origin of the main sources of indoor particulate matter emitted during permanent home occupancy, as well as a comparison of the responses of different aerosol monitors. Tracking the basic sources of the generation of airborne particles may allow the development of optimal living conditions and habitant’s customs that would minimize PM exposure. The low-cost air sensors can be an important source for the identification of specific air pollution sources and selection of optimal and properly configured air purification systems. 

Recent reports revealed a significant relationship between PM levels and the severity of COVID-19 symptoms. Prolonged exposure to a high PM_2.5_ and PM_10_ can promote the overexpression of alveolar ACE-2 receptors which are responsible for binding of the virus to the tissue. This was linked to the infection severity for the patients exposed to elevated PM levels [33,34,35]. It was also suggested that viruses can be carried on the PM surface [36]. Therefore, airborne PM detection and air purification can have implications for virus transmission, course of the disease, and long-term side effects of COVID-19.

The experiments were divided into two campaigns comparing particle concentrations in a permanently occupied house in high- and low-occupancy rooms.

## 2. Materials and Methods

### 2.1. The Location and Building Characteristics

The experiments were carried out in the Bielany region in Warsaw in a nine-floor building (built in 2012) with three flats on the floor. The experimental flat was on the fourth floor. Its overall volume was about 90 m^2^ and the ceiling height was 2.7 m. The flat was occupied almost permanently by a four-person family and a small dog due to the COVID-19 lockdown. The flat’s ventilation was mechanically supported by a common building air pump system. During the experiments, the householders led a normal life, although they spent almost the entire time at home—mostly in the living room.

### 2.2. Experimental Setup and Data Collection

High-occupancy room

In the first campaign (28 March 12:00 a.m.–1 April 11:59 p.m.), measurements were performed in the living room (21 m^2^) which was nearly permanently occupied, from 8:00 a.m. to 1:00 a.m. (about 16–17 h per day), by up to four persons. Most of the activities were related to remote work and school duties performed by the children. The kitchen (8 m^2^) was directly connected to the living room; thus, all meal production activities had a pronounced impact on air quality. Two air monitors based on light scattering were employed for the measurements: an AeroTrak8220 (TSI INC., Shoreview, MN, USA) and an OPC-N3 (AlphaSense Ltd., Great Notley, UK). The first was used as a reference instrument. The AeroTrak 8220 and OPC-N3 were placed about 60 cm above floor level, which corresponded to the head level of a person sitting on a sofa. The inlets of the devices were placed about 15 cm from each other to collect air from a similar area. The measurement data were recorded in the AeroTrak8220 internal memory, while the OPC-N3 was connected to the computer running the manufacturer’s software (Version OPC 1.0.6457.18465), allowing data visualization and recording in a CSV file. The activities such as “window”, “cooking/frying/toast/popcorn”, “vacuuming”, and “sport” were manually noted and then plotted on the graph as corresponding single marks on a timeline. The time of the activity beginning was noted.

Low-occupancy room

**Figure 1:**
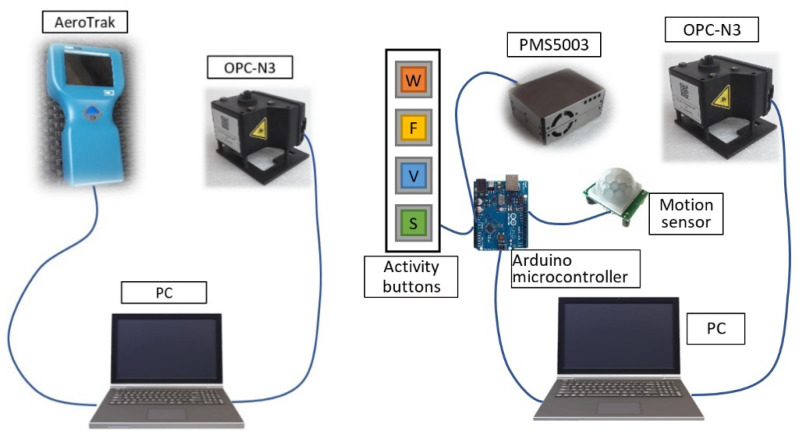
Experimental setups used in the first campaign (**left**) and in the second campaign (**right**).

In the second campaign, running from 25 April 12:00 a.m.–5 May 11:59 p.m., the tests were conducted in a room (15 m^2^) going off the kitchen and the living room. It was occupied mostly by one teenager and a small dog. The room was separated from the kitchen and living room by about a 5 m long hall (12 m^2^). The daytime activities in the room were related mostly to educational tasks. The visits of other residents were occasional, mainly for the supervision of the aerosol measurement setup, advice during lessons, and evening exercises (up to three persons). Due to the noisy operation of the AeroTrak8220, the device was excluded in the campaign. To obtain results that are more useful and comprehensive, two low-cost sensors were used—an OPC-N3 (AlphaSense) and a PMS5003 (Plantower, Beijing, China). Both sensors apply very small fans to draw air/particles into the interrogating laser beam. The airflow method applied facilitates a near-noiseless operation of the sensors. Both devices were mounted about 70 cm above floor level, which corresponded to the average head position when sitting at the desk and sleeping on the bed. On the basis of previous campaign experiences, the measurement and data recording setup was improved to obtain an automatized correlation between the sensor’s readings and the actual activity. The power supply and data acquisition process of OPC-N3 was as previously described. The PMS5003 sensor was controlled by means of the Arduino platform. The setup was coupled with a PIR (Passive Infra Red) motion sensor to get real, automatic tagging corresponding to the movement timeline (“movement”). Since some other activities were known to influence the particle concentration, the setup was equipped with four LED touch buttons: “window”, “frying”, “vacuuming”, and “sport”, which were activated by the user according to the action taken (Figure 1). In this campaign, frying and toast-making was not differentiated due to the electronic device’s limitations. The activated buttons marked the on/off state to the user with an LED (light-emitting diode). The time-correlated data with proper activity stamps were recorded in an ASCII file. The activity record button was switched on when the activity was started and switched off when it was finished. During the second campaign, the movement restrictions were gradually decreased. However, there were only two days (25 April and 1 May) when all residents left the apartment for several hours. During other days, the habitants stayed at home as during the first campaign. 

In both campaigns, a commercial air purifier PHILIPS model AC3259 (PHILIPS, Drachten, The Netherlands) was used. The effectiveness in PM removal was compared by intentionally switching the device on and off during some periods. 

### 2.3. Statistical Methods

All statistical analysis was performed using OriginPro 9.1 (OriginLab Corporation, Northampton, MA, USA). The mean and standard deviation were calculated using the descriptive statistic module on a specified data range. The correlation coefficients were calculated to compare performance between the instruments. Pearson’s *r* coefficient value used to estimate the linearity between readings of the sensors. The linear regression slope between readings showed the detection performance ratio between the sensors. A two-sample *t*-test was used to estimate the differences between the means of two independent results. The tests were performed assuming a 95% confidence level. 

## 3. Results and Discussion

### 3.1. Dependence of PN and PM Concentrations on the Resident’s Activities in a Highly Occupied Room 

**Figure 2:**
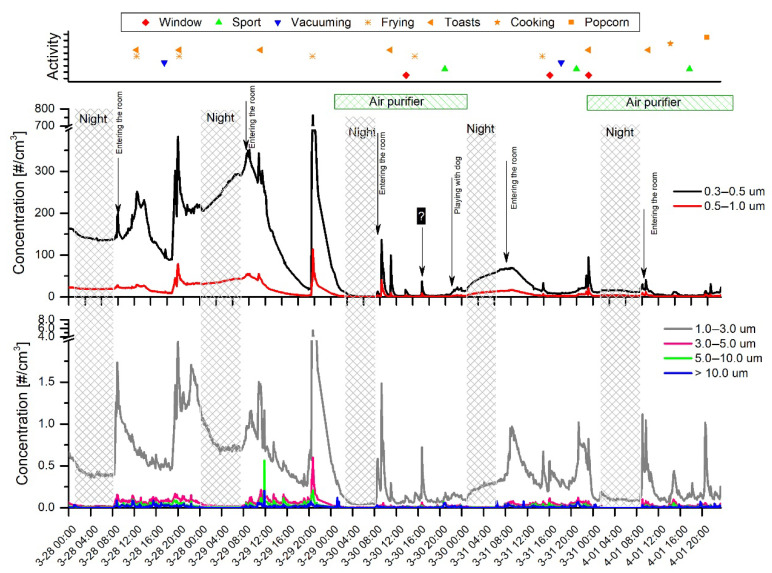
Time series of particle number (PN) concentrations recorded with the AeroTrak 8220 (TSI). The horizontal green bars represent the periods of air purifier usage in the room.

**Figure 3:**
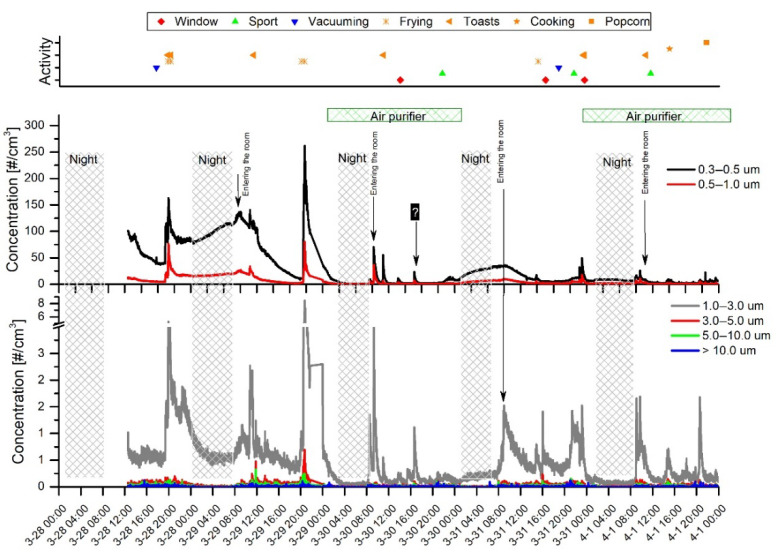
Time series of PN concentrations recorded with the OPC-N3 (AlphaSense). The horizontal green bars represent the periods of air purifier usage in the room.

The daily fluctuations of particle number (PN) and mass (PM) concentrations showed a periodic pattern with apparent decreases and curve flattening during the night and elevated levels in the daytime. Figure 2 and Figure 3 show the PN concentration time series obtained using the AeroTrak8220 and AlphaSense OPC-N3, respectively. For clarity reasons, the measured particle size bin ranges were split into separate subplots. The OPC-N3 measurements started a few hours later due to data storage problems. In both figures, the nighttime periods were labeled corresponding to the moment of leaving the room by the last resident in the night and the first entering the room in the morning. The strong increase in PN was observed just at the beginning of the morning activities. It produced distinct spikes, particularly when doors were opened vigorously; however, gentle door opening also resulted in apparently elevated PN concentrations. It is worth noting that, during the whole experimental period, the minimum particle PN concentrations recorded with the AeroTrak8220 were 1.12, 0.31, and 0.03 #/cm^3^ in 0.3–0.5, 0.5–1.0, and 1.0–3.0 µm bins, respectively. This shows that the submicron and fine particles were permanently suspended in the air. On the other hand, the particles >3 µm tended to settle, and we observed multiple records with no particles detected, especially in the night and during the periods of air purifier operation. 

Multiple studies have revealed that air filtration is an effective method to reduce exposure to indoor aerosols and bioaerosols [37,38,39]. Table 1 shows average PN concentrations recorded with both air monitors for the data grouped according to day, night, and air purifier on and off periods. One could observe about 10 times lower PN concentrations when the air purifier was operating. Due to the large PN fluctuations, the standard deviation of the data was comparable to or even higher than the mean values. However, the two-sample *t*-test showed that differences were statistically significant at a 95% confidence level. 

Surprising results were obtained for the nighttime measurements revealing higher average PN concentrations than in the daytime. The results obtained seem inconsistent with those intuitively expected, as well as those reported in the literature [40,41,42]. However, the discrepancy between expected and actual readings can be attributed to the infiltration of outdoor particles, which most probably penetrated the room through the vent holes in the windows [43,44,45,46]. The particularly high PN levels recorded on 28–29 March, as well as 31 March–1 April, were derived from extremely high PM_2.5_ and PM_10_ concentrations reported for Warsaw. The data are in agreement with the readings available for the nearest air monitoring station at Tołstoja 2 Str. (International code L0308A) [47].

The experimental data clearly demonstrated that meal preparation such as frying, making toast, and popcorn microwaving belonged to the major particle sources which produced peak concentrations up to 882 and 350 #/cm^3^ when measured with the AeroTrak 8820 and OPC-N3, respectively. During frying, the PN concentration rose rapidly within a few minutes, while the decay to the background concentration was prolonged up to 4–8 h. The highest increases related to frying were observed for particles in a range of 0.3–3 µm. Moderate PN increases were usually observed for particles >3 µm. However, on 29 March frying and toast-making resulted in a distinctly elevated concentration of particles >3 µm. One can conclude that the appearance of coarse particles was observed during high-intensity frying. While boiling is a long-term process compared to frying, it was not identified as a significant activity increasing indoor air PN and PM concentrations. Particle emissions during soup boiling (1 April about 2:00 p.m.) were some of the lowest identified among other activities. A slight increase in the number of particles sized 0.3–0.5 µm and a more distinct peak of 1.0–3.0 µm particles were observed.

Various studies support the findings of this work that the particle emissions during cooking heavily depend on the type of meal and preparation method. For example, frying generates more particles than boiling [15,48,49].

#### 3.1.1. The Influence of Residents’ Activities on PM_1_, PM_2.5_, and PM_10_ Concentrations in a Highly Occupied Room 

The OPC-N3 built-in algorithm allows estimation of PM concentrations as a function of particle number concentration in corresponding particle diameter bins, with the assumption of an average particle density of 1.65 g/cm^3^. The PM values are determined according to European Standard EN 481. However, there is no comprehensive information in the literature on applied PM calculation algorithms [50,51]. 

**Figure 4:**
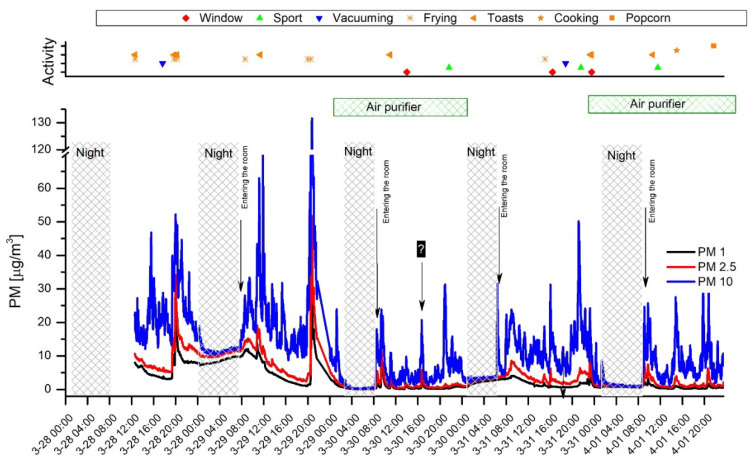
The particulate matter (PM_1_, PM_2.5_, and PM_10_) timeline records obtained with the OPC-N3. The horizontal green bars represent the periods when the air purifier was used in the room.

The PM concentration records obtained with the OPC-N3 are presented in Figure 4. The peak values observed during various activities are consistent with the PN readings presented in Figure 2 and Figure 3. However, some activities such as exercise and vacuuming were more distinctly reflected when PM_10_ records were considered. This shows that larger particles have pronounced input in mass concentration. They appear in the air due to resuspension when mechanical forces and air agitation occur [18,52].

The average PM_1_, PM_2.5_, and PM_10_ values presented in Table 2 demonstrate that the PM concentrations were considerably lower in periods of air purifier operation. The PM reduction efficiency depended on the particle fraction size resulting in a decrease in PM_1_, PM_2.5_, and PM_10_ of 7.13, 5.20, and 3.05 times, respectively. The higher effectiveness of fine particle removal positively contributes to the reduction in possible adverse health effects related to inhalable particles. As could be expected, both PM_2.5_ and PM_10_ levels were lower during the nighttime compared to the daytime. The more distinct PM_10_ decrease can be explained by the absence of coarse particles during the nighttime when air movement was suspended. On the other hand, the PM_1_ concentration during nighttime was higher compared to the daytime. This result is in agreement with findings described in the previous section showing an elevated PN due to particularly high outdoor particle concentrations. It also supports the fact that fine particles do not tend to settle and are generally present even in immobile ambient air, at least for a period of a few hours.

The obtained average PM_2.5_ (4.53 ± 5.00 µg/m^3^) and PM_10_ (11.48 ± 11.78 µg/m^3^) concentrations are optimistic since they are below the established in European Commission Air Quality Standards [53], values which are 25 and 50 µg/m^3^, respectively. However, the data do not cover the autumn/winter period which is known for the highest PM levels. Moreover, the mean PM_10_ in Warsaw was considerably lower than the PM_4_ measured in the one of the most polluted regions in Poland—Silesia (27.89 ± 4.74 µg/m^3^) [54].

#### 3.1.2. Comparison of PN Concentrations Measured with OPC-N3 and AeroTrak8220

**Figure 5:**
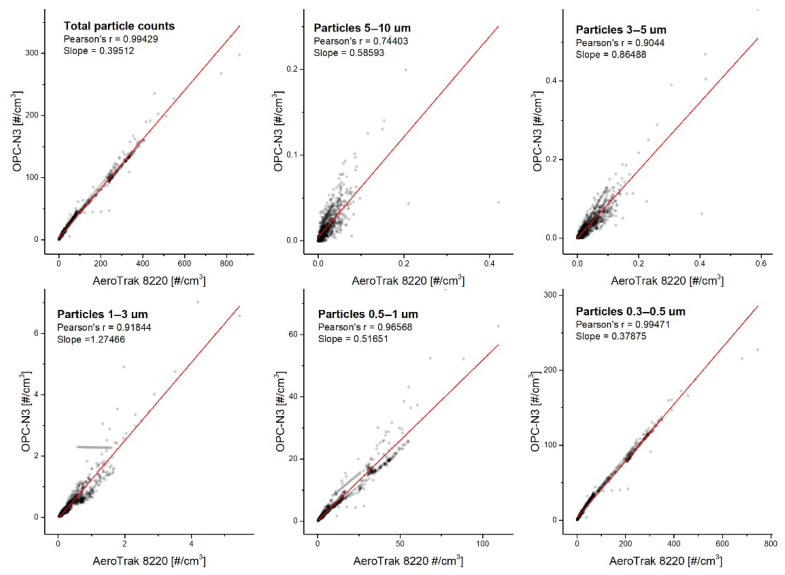
The correlation of particle number concentration in corresponding size bins recorded with the AeroTrak8220 and OPC-N3.

At the same time and under identical ambient conditions, the PN concentration time series reported by the AeroTrak8220 and OPC-N3 displayed a similar pattern. However, the AlphaSense monitor appeared to underestimate the total particle concentration readings. The mean PN concentrations of all detected particles during the experimental period was 95.52 ± 114.10 #/cm^3^ and 37.68 ± 46.29 #/cm^3^ for the AeroTrak8220 and OPC-N3, respectively. The lower counting efficiency of the previous AlphaSense model (OPC-N2), especially in low particle concentrations, was previously reported [28,55]. One of the reasons contributing to the underestimation of the results concerns the 50% efficiency of counting particles of 0.3 µm diameter [51]. Figure 5 shows the correlations of both the AeroTrak8220 and the OPC-N3 readings with respect to the fitted bin classification. The slopes of the linear regression obtained in most cases were within the range 0.38–0.86, indicating that the OPC-N3 underestimates the PN concentration compared to the AeroTrak8220. The only exception was observed for 1–3 µm particles, whose correlation slope was 1.27, demonstrating that the OPC-N3 slightly overestimates the particle counts in this range. 

### 3.2. Dependence of PM Concentrations on the Residents’ Activities in the Low-Occupancy Room

**Figure 6:**
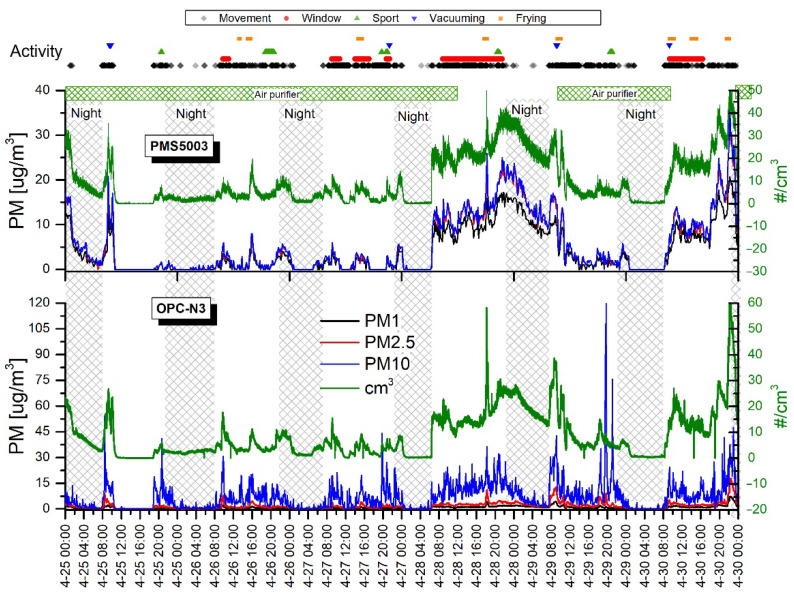
Variations in indoor mass concentrations of PM_1_, PM_2.5_, and PM_10_ measured with the PMS5003 and OPC-N3 sensors for the first week. The green linear graph shows total particle counts for each sensor (right axis). The activity points on the top are common to both sensor graphs.

**Figure 7:**
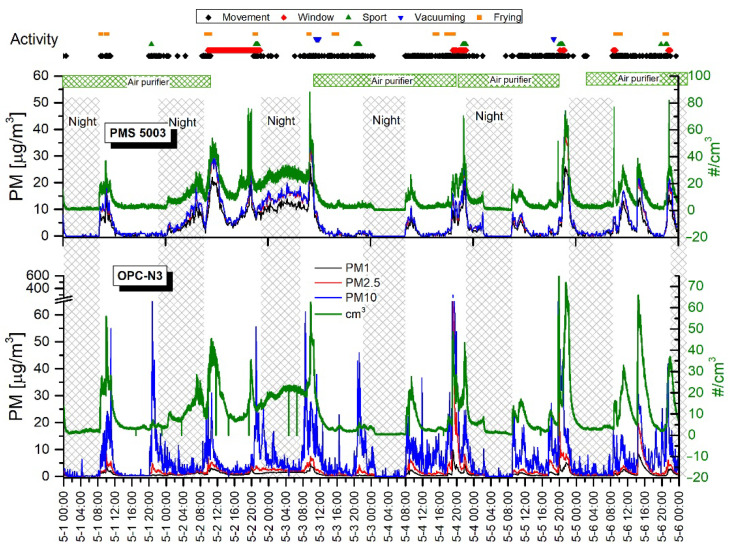
Variations in indoor mass concentrations of PM_1_, PM_2.5_, and PM_10_ measured with the PMS5003 and OPC-N3 sensors for the second week. The green linear graph shows total particle counts for each sensor (right axis). The activity points on the top are common to both sensor graphs.

Figure 6 and Figure 7 show the time series of PM concentrations and the total particle counts recorded with two sensors, the OPC-N3 and PMS5003 (Plantower), from 25 April–5 May. The shadowed vertical nighttime areas represent roughly the hours corresponding to the main room’s occupants’ sleep time. The upper part of the graphs shows time-correlated activities that potentially have an impact on PM levels.

The mean PM_1_, PM_2.5_, and PM_10_ concentrations recorded for daytime and nighttime with OPC-N3 are presented in Table 3. This shows lower PM levels at night than at daytime. A bigger particle fraction denotes a more distinct decrease in PM concentrations: PM_10_ > PM_2.5_ > PM_1_. This pattern can be explained by the lower sedimentation rate of smaller fractions; thus, differences between day and nighttime are not distinct. Analogously, the bigger particles deposited on the surface during the nighttime after daytime air agitation rise and strongly contribute to PM10.

The black diamonds in Figure 6 and Figure 7 represent motion events regardless of the number of persons and their activity. This clearly shows that the elevated PM and PN concentrations occurred when the motion events were recorded. The nighttime concentration curve flattening was delayed due to temporal movements and the lag time of particle descent. As in the previous research campaign (measurements in the highly occupied room), morning activities, such as entering the room, always produced a steep increase in particle concentrations. Table 4 shows the mean PM concentrations calculated for motion and motionless periods. It clearly demonstrates that motion contributes to increased PM levels. It is worth noting that, when motion disappeared, the particles were still suspended in the air for hours before the plateau was reached. Therefore, the differences between average concentrations are not spectacular, although they are statistically significant.

The periods of motionlessness took place on 25 April (10:00 a.m.–7:00 p.m.) and on 1 May (12:00 a.m.–8:00 p.m.) when all residents left the apartment for several hours. One can observe that PM and PN concentrations reached even lower levels than were usually observed at nighttime. For example, on 25 April (11:30 a.m.–18:30 p.m.), when the plateau was reached, the mean PM_10_ measured with OPC-N3 was 0.03 ± 0.12 µg/m^3^ and the maximum value was 1.63 µg/m^3^. Thus, over 150 times lower PM_10_ concentrations were produced compared to the average PM_10_ achieved during the nighttime when only one sleeping person occupied the room. This demonstrates that each motion or even the presence of the sleeping person in the room generated particles. Another motionless daytime was on 2 May (10.30 a.m.–8.30 p.m.). However, in contrast to the previous observation, a major increase and fluctuations in PM and PN concentrations occurred. This could be attributed to the influence of outdoor air due to the opened window (red dots), as well as a break in air purifier operation. These two factors could concurrently contribute to the variations observed in aerosol concentrations.

The elevated PM levels during sports activity (green triangles) were recorded mainly by the OPC-N3, while the PMS5003 in some cases did not show specific changes in PM and PN concentrations. The most noticeable increases (about 2–10 times higher compared to the period before the exercises started) were observed for PM_10_ values, while PM_2.5_ and PM_1_ changed imperceptibly. The increases in concentrations can be attributed to the dust resuspension from the surfaces and particles detaching from clothes. Compared to the previously described campaign, the sports activity in the low-occupancy room had a visibly higher impact on particle levels. Several factors can explain the discrepancy between strength of sensor response and the sports activity. These factors include (1) differences between the volume of the rooms—in a smaller room the particle source can produce a relatively higher PN, and (2) a lower background level in the low-occupancy room due to the lower air agitation rate where each movement can, thus, entrain and resuspend the particles. 

Similarly, as in the previous campaign, meal preparation such as frying and toast-making had a prominent impact on particle concentrations. Both the OPC-N3 and the PMS5003 indicated high and sharp peaks corresponding to frying intensity. However, the relatively long distance between the kitchen and the room attenuated the particle cloud and, thus, the measured values were not so distinct as previously. Moreover, the peak occurrence was delayed for about 5 min from the beginning of the frying action. The particularly high peaks observed on 28 (5:45 p.m.) and 30 April (9:00 p.m.) were related to burning the dish on a pan and in the oven, respectively.

The indoor aerosol concentrations can heavily depend on the outdoor levels. In this campaign, the outdoor concentrations were not measured and, therefore, the influence of outdoor particles on concentrations measured in the room was difficult to estimate. According to the window opening labels (red dots), there were observed elevated PM and PN concentrations measured with both sensors when the window was opened. 

Due to the superimposed periods of vacuuming and other activities such as an opened window, frying, and movement, it was difficult to judge the influence of vacuuming. Slightly elevated PM_10_ levels, which could be attributed to vacuuming, were detected with the OPC-N3 sensor. In general, a moderate influence of vacuuming on PM levels was observed. On the other hand, several studies have shown that, while vacuum cleaning contributes to the temporal increases in particle emissions, there is no rule allowing determination of the significance of the source. It has been found that multiple factors can influence particle emissions including room vacuuming (frequency, quality, and age and type of vacuum cleaner), presence of carpets on the floor, and the HEPA (High-efficiency particulate air) filter used [56]. 

Table 5 shows the average values obtained with the sensors when the Air purifier was on and off. It clearly shows a remarkable (2–3 times) reduction in PM and PN levels while the air purifier was operating. Relatively steep decreases in particle counts were observed from the beginning of the purifier’s work (Figure 6) (9:00 a.m. 29 April) and (Figure 7) (2:00 a.m. 5 May). It should be noted that almost flat particle concentration levels were obtained during the nighttime when the air purifier was working. The two-sample *t*-test showed that statistically significant differences of mean particle mass and number concentrations were obtained when the air purifier was on or off. 

#### Comparison of PMS5003 and OPC-N3 Readings in the Low-Occupancy Room

Both the PMS5003 and the OPC-N3 sensors allow readings of PM_1_, PM_2.5_, and PM_10_ levels. In general, the two devices clearly responded to increases and decreases in PM concentrations, although the readings were different. The average PM_10_ levels measured by the OPC-N3 were about 20% higher compared to the PMS5003. Contrary to this, the PMS5003 showed about 470% and 280% higher average PM_1_ and PM_2.5_, respectively, than the OPC-N3 (Table 6). According to literature evaluation studies, AlphaSense shows a good correlation with reference devices even though the PM readings were found to be underestimated [57,58,59]. On the other hand, a machine learning algorithm can effectively improve the calibration and performance of low-cost optical sensors that could be used in the future [60]. 

Surprisingly, the PM and PN concentration responses of the PMS5003 had a nearly identical shape, which was also very similar to the PN recorded with the OPC-N3 (Figure 6 and Figure 7). This suggests that the PM concentration in PMS5003 is calculated regardless of particle size. The average PN concentration measured with PMS5003 and OPC-N3 showed very close values of 10.24 ± 11.01 and 9.44 ± 9.95 µg/m^3^, respectively.

**Figure 8:**
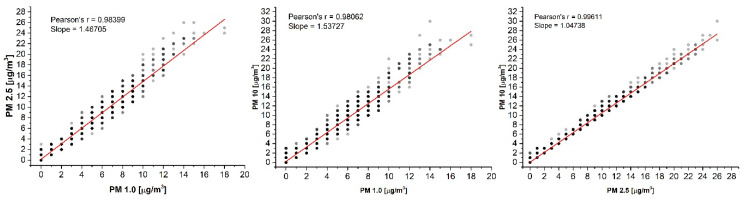
Correlation among the PM readings of the PMS5003 monitor. The data represent all measurements for one representative day. Similar results were obtained for the remaining single days, as well for the whole set of data.

Figure 8 shows PM-wise sets of PM_1_–PM_2.5_, PM_1_–PM_10_, and PM_2.5_–PM_10_ readings obtained with the PMS5003 sensor. A regression analysis revealed a very high correlation resulting in Pearson’s *r* coefficients close to one. An almost linear relationship among PM_1_, PM_2.5_, and PM_10_ is surprising since the indoor particle size distribution and number can vary depending on many factors such as aerosol source, motion, and outdoor concentrations. The near-linear relationship among PMs could take place only at ideal ratios of particle size and number, which would be difficult to achieve. A possible explanation of such a highly linear response is the bulk sample measurement despite individual particles. The ambient air does not directly pass a laser beam but is drawn through a sinuous channel, which does not favor measuring single particles and precisely evaluating their size. 

**Figure 9:**
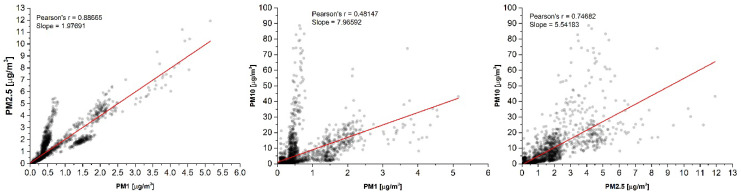
Correlation among PM readings of the AlphaSense OPC-N3 monitor. The data represent all measurements for one representative day. Similar results were obtained for the remaining single days, as well for the whole set of data.

A different pattern was observed in the PM-wise comparison of the OPC-N3 readings. The correlation factors calculated indicate the considerable diversity of certain PM readings that showed a diverse and multimodal distribution between certain PMs (Figure 9).

### 3.3. Comparison of PM in the High- and Low-Occupancy Rooms

**Figure 10:**
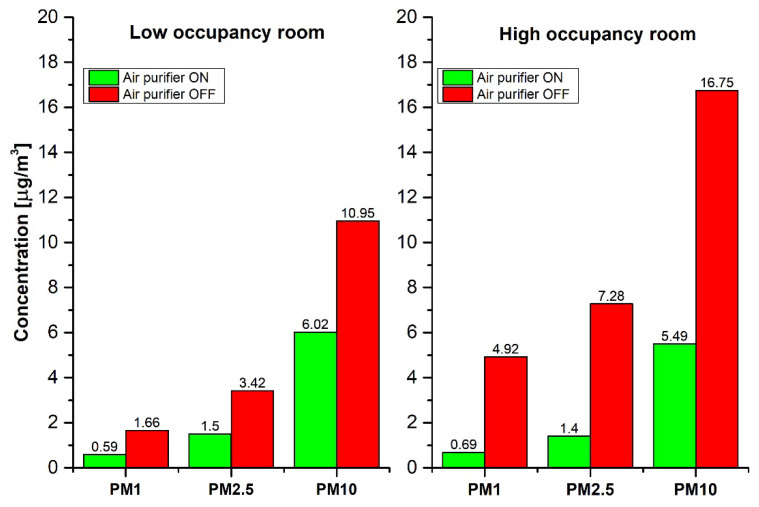
The mean PM concentrations recorded with OPC-N3 in the low- and high-occupancy rooms when the air purifier was operating or not. The correlations were statistically significant at the 95% confidence level.

During the time the air purifier was not working, the PM_1_, PM_2.5_, and PM_10_ concentrations in the high-occupancy room were distinctly elevated compared to the low-occupancy room (Figure 10). This shows that the number of persons and the intensity of room usage had an impact on PM levels. In addition to the occupancy, the close vicinity of the kitchen could substantially contribute to the increased PM levels. In contrast, when the air purifier was operating, the PM concentrations in both rooms were almost the same. This was most probably due to the feedback motor control when the air purifier’s integrated dust sensor detected increased aerosol levels.

## 4. Conclusions

This study presented a comparison of low-cost sensor responses to an aerosol generated by domestic activities during permanent home occupancy. However, the presented research was not intended for generalization to all cases. The study was limited to one apartment and a specific period related to the restrictions imposed due to the COVID-19 lockdown. Initially, the research plans included larger-scale experiments but the dynamic changes in the restrictions were difficult to predict. At that time, it was possible to deploy only one measurement setup in one flat only. It was expected that the only opportunity to perform experiments was during a deep lockdown; therefore, the research area was narrowed to the data obtained in such unique conditions. Another limitation of the study stems from the measurement range of the sensors, which could not measure particles under 0.3 µm. Therefore, the total particle number and mass concentrations could have been underestimated compared to the sample weighting collection methods.

Due to the lockdown, it was possible to conduct these studies continuously over the entire time. All the activities could be supervised and tracked precisely, which is usually difficult when residents are required to control and log their activities. The automatic motion control and feasible function tagging synchronized with the sensor readings improved and facilitated data tracking and analysis to a great extent.

One of the achievements of this research was a performance comparison of three OPCs. The sensors showed different levels of particle counting efficiency as follows: AeroTrak8220 > OPC-N3 > PMS5003. This is in correspondence with the effectiveness of the air intake systems of these sensors and the optical system used, as reflected in the price of the sensor. Another important finding was that indoor aerosol concentrations in the low-usage room were considerably lower than in the room located in close vicinity to the kitchen, occupied by four persons. For this reason, the simplest way to reduce the inhalation dose seems to be staying in separate rooms. The last suggestion should of course consider sociological issues that are especially important during an imposed confinement in the home. 

The studies clearly showed that an air purifier most effectively reduced and controlled indoor aerosol concentrations. Therefore, its use in occupied houses is fully justified. Conversely, window-opening contributed to increased aerosol levels; hence, ventilation should be recommended only if the outdoor aerosol concentrations are lower than the indoor.

Among indoor air contaminants, kitchen activities such as frying and toast-making were the most powerful sources of indoor air pollution, characterized by particles of mainly <3 µm. The hazardous role of air pollutants which originate from the kitchen may increase due to prolonged exposure, which can be particularly strong in home bureaus where the room and kitchen are organized in a common space. In addition to kitchen fumes, vigorous movement such as physical exercise produces substantial amounts of particles that originate mainly from dust layer resuspension, resulting in the appearance of a fraction of coarse particles.

Lastly, considering the efficiency of the low-cost sensors examined, as well as the comfort of noiseless operation, the OPC-N3 was especially effective in accurately tracking indoor aerosol variations, and, when coupled with automatic event tagging, it can provide useful information for air quality management and the exact estimation of personal PM exposure during the occupancy period.

## Figures and Tables

**Table 1:** The mean PN concentrations were recorded with OPC-N3 and AeroTrak8220 sensors. The asterisks (*) denote the pairs with a statistically significant difference confirmed with a two-sample *t*-test at the 95% confidence level. Only the pair day–night recorded with the AeroTrak8220 instrument did not reveal a statistically significant difference of the mean. The day and night data include the air purifier on or off periods and vice versa.

	Day (#/cm^3^)	Night (#/cm^3^)	Air Purifier On (#/cm^3^)	Air Purifier Off (#/cm^3^)
OPC-N3	38.09 ± 50.59 *	46.45 ± 53.59 *	7.51 ± 7.94 **	68.77 ± 56.25 **
AeroTrak 8220	82.63 ± 115.75	97.28 ± 121.11	13.61 ± 15.86 ***	155.75 ± 116.98 ***

**Table 2:** Average PM concentrations recorded with the OPC-N3. The letters a–g show pairs for which statistical significance test was performed. The *t*-test showed that results were statistically significant at the 95% confidence level.

	Day (µg/m^3^)	Night (µg/m^3^)	Air Purifier On (µg/m^3^)	Air Purifier Off (µg/m^3^)	Overall Average PM Concentration (µg/m^3^)
PM_1_	2.86 ± 3.57 ^a^	3.18 ± 3.51 ^a^	0.69 ± 0.80 ^d^	4.92 ± 3.86 ^d^	2.94 ± 3.56
PM_2.5_	4.71 ± 5.21 ^b^	3.96 ± 4.22 ^b^	1.40 ± 1.46 ^e^	7.28 ± 5.38 ^e^	4.53 ± 5.00
PM_10_	13.65 ± 12.50 ^c^	4.67 ± 4.77 ^c^	5.49 ± 5.87 ^f^	16.75 ± 13.08 ^g^	11.48 ± 11.78

**Table 3:** Mean PM concentrations recorded with the OPC-N3 during the nighttime and daytime. The *t*-test showed that results were statistically significant at the 95% confidence level.

	Night	Day
PM_1.0_ (µg/m^3^)	0.59 ± 0.74	0.99 ± 1.08
PM_2.5_ (µg/m^3^)	1.03 ± 1.43	2.54 ± 4.32
PM_10_ (µg/m^3^)	3.13 ± 4.74	9.93 ± 16.23

**Table 4:** Mean PM concentrations recorded with the PMS5003 and OPC-N3 for the periods when the motion was detected or not. The *t*-test showed that results were statistically significant at the 95% confidence level.

		Motion	No Motion
PM_1.0_ (µg/m^3^)	PMS5003	4.31 ± 4.70	3.86 ± 4.98
	OPC-N3	1.07 ± 1.10	0.81 ± 0.96
PM_2.5_ (µg/m^3^)	PMS5003	5.96 ± 6.37	5.26 ± 6.71
	OPC-N3	3.23 ± 6.30	1.83 ± 3.24
PM_10_ (µg/m^3^)	PMS5003	6.25 ± 6.72	5.49 ± 7.05
	OPC-N3	14.13 ± 24.58	6.65 ± 12.01

**Table 5:** Average PM and PN concentrations when the air purifier was operating.

		Air Purifier On			Air Purifier Off		
		Mean	SD	Max	Mean	SD	Max
Counts (#/cm^3^)	PMS5003	2.95	4.44	43.00	25.59	9.71	88.00
	OPC-N3	6.49	7.56	65.88	20.23	10.20	79.47
PM_1_ (µg/m^3^)	PMS5003	6.03	6.79	70.92	10.91	4.19	32.00
	OPC-N3	0.59	0.82	10.45	1.66	1.05	14.51
PM_2.5_ (µg/m^3^)	PMS5003	1.96	3.06	27.00	14.42	6.10	43.00
	OPC-N3	1.50	3.40	98.74	3.42	3.45	134.89
PM_10_ (µg/m^3^)	PMS5003	2.80	4.23	36.00	14.98	6.68	52.00
	OPC-N3	6.02	13.02	579.09	10.95	13.02	7.93

**Table 6:** Average, minimum, and maximum concentrations of PM_1_, PM_2.5_, and PM_10_ obtained during the experimental campaign with the PMS5003 and OPC-N3.

	OPC-N3		PMS5003	
	Average PM Concentration (µg/m^3^)	Min/Max Concentration (µg/m^3^)	Average PM Concentration (µg/m^3^)	Min/Max Concentration (µg/m^3^)
PM_1_	0.82 ± 0.97	0/14.51	3.88 ± 4.97	0/32
PM_2.5_	1.91 ± 3.5	0/134.89	5.29 ± 6.70	0/43
PM_10_	7.08 ± 13.18	0/602.2	5.53 ± 7.05	0/52
#/cm^3^	9.44 ± 9.95	0/79.47	10.24 ± 11.01	0/88
